# Chylothorax as an Initial Manifestation of Waldenström macroglobulinemia

**DOI:** 10.7759/cureus.7566

**Published:** 2020-04-06

**Authors:** Natalia Rodriguez Botero, Andres Zerrate Misas, Kenny Mauricio Galvez Cardenas, Juan David Ramirez Quintero

**Affiliations:** 1 Internal Medicine, Universidad Pontificia Bolivariana, Medellin, COL; 2 Thoracic Surgery, Hospital Pablo Tobon Uribe, Medellin, COL; 3 Hematology, Hospital Pablo Tobon Uribe, Medellín, COL; 4 Internal Medicine, Hospital Pablo Tobon Uribe, Medellín, COL

**Keywords:** waldenström macroglobulinemia, chylothorax

## Abstract

Waldenström macroglobulinemia (WM) is an uncommon disease whose most common presenting features are anemia, hyperviscosity-related symptoms, B symptoms, bleeding, and neurological symptoms. Pulmonary compromise is rare, and there are a few cases reported of chylothorax as a manifestation of Waldenström macroglobulinemia. We present the case of a patient who presented with a refractory chylothorax as the initial manifestation of Waldenström macroglobulinemia.

## Introduction

Waldenström macroglobulinemia (WM) is a lymphoplasmacytic lymphoma (LPL) associated with a monoclonal immunoglobulin M (IgM) protein and belongs to the category of non-Hodgkin B lymphomas with an indolent course. The diagnosis of WM is based on the histopathological confirmation of bone marrow (BM) infiltration by lymphoplasmacytic cells/LPL and the detection of any amount of monoclonal IgM protein [1]. The clinical manifestations of WM develop secondary to direct tumor infiltration and/or effects of IgM monoclonal protein in the blood [2]. At least 25% of patients with WM are asymptomatic at diagnosis. The initial symptoms are non-specific and include fatigue, fever, malaise, and weight loss [3]. Pulmonary manifestations represent only 3-5% of cases [4]. Chylothorax is a very uncommon presentation, with only nine cases reported to date in the literature.

## Case presentation

**Figure 1:**
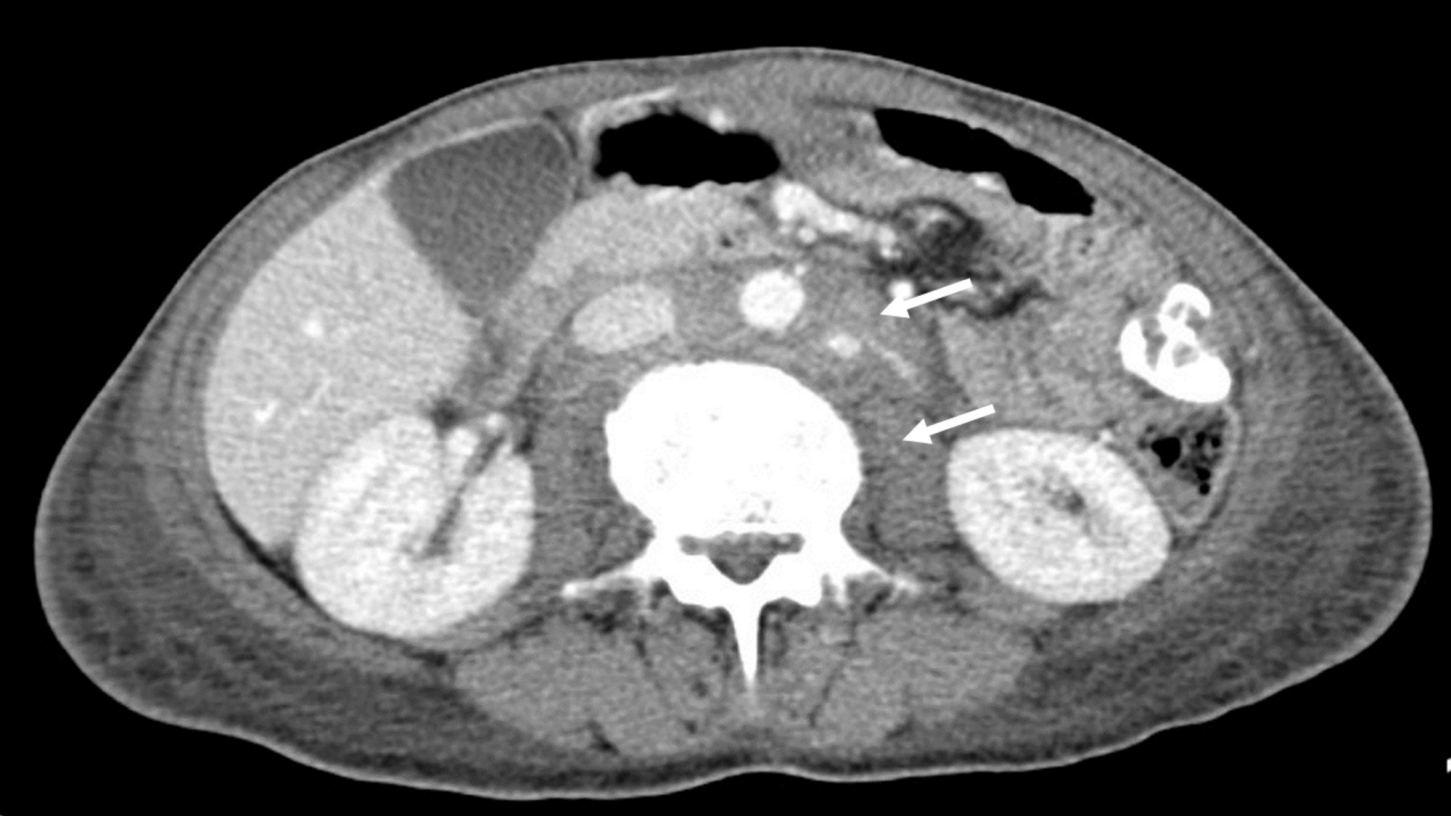
CT of the abdomen showing conglomerate of adenopathies

A 63-year-old female with no significant medical history presented with a three-month complain of cough, shortness of breath, pleuritic chest pain, and weight loss. Initially, she was admitted to another hospital where a chest X-ray was performed that showed a left pleural effusion. They placed a thoracostomy tube, and the study of the fluid revealed triglyceride levels of >300 mg/dL, which confirm the diagnosis of chylothorax. The patient was taken to pleurectomy and decortication by thoracoscopy but showed no improvement; therefore, she was admitted to our hospital. Her vital signs were normal, and the physical examination was remarkable for cervical and axillary lymphadenopathy. Initial tests showed moderate anemia, lymphopenia, and thrombocytosis; kidney and liver function were normal. Medical treatment for the chylothorax was initiated with low-fat diet and total parenteral nutrition. A CT scan of the thorax and abdomen showed lymphadenopathies at the base of the neck and in both axillary pits, and conglomerate of adenopathies that encompassed the aorta, the vena cava, the origin of the celiac trunk, the origin of the mesenteric artery, and the renal vessels, without mediastinal adenopathies, hepatosplenomegaly or lung masses, with imaging being highly suspicious of lymphoma with involvement on both sides of the diaphragm (Figure 1).

**Figure 2:**
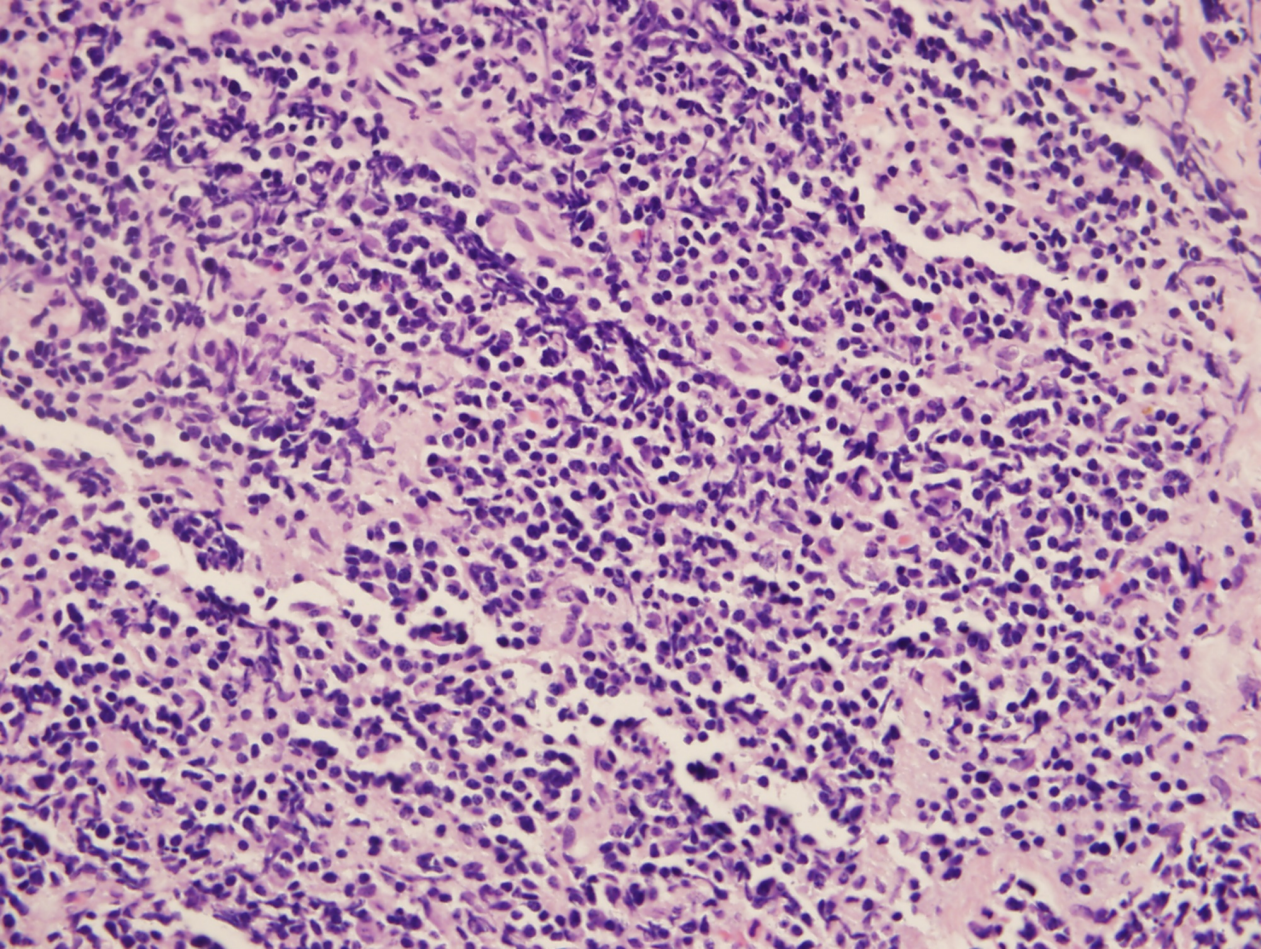
Bone marrow biopsy (hematoxylin and eosin stain)

**Figure 3:**
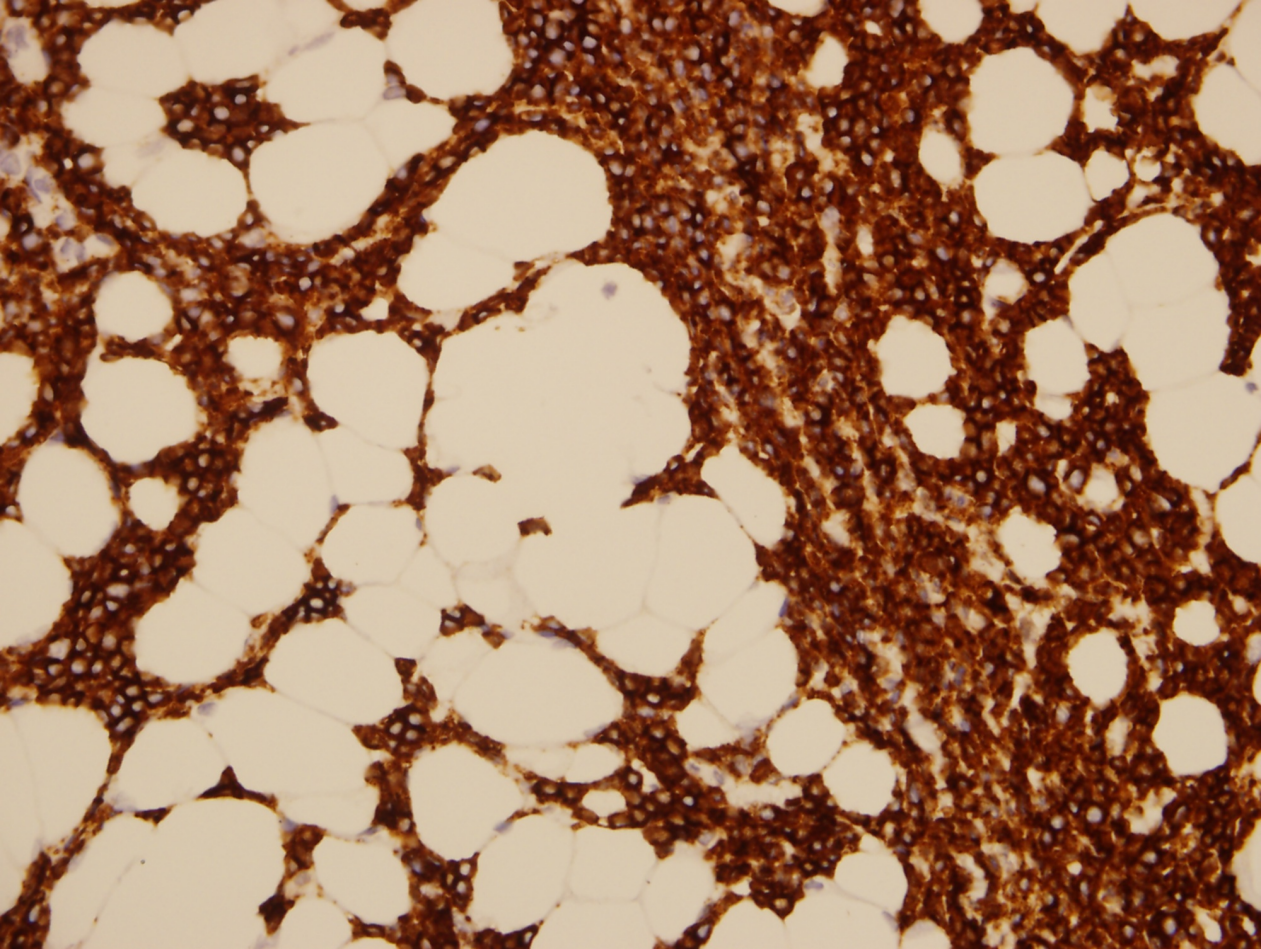
Bone marrow biopsy (CD20 marker)

**Figure 4:**
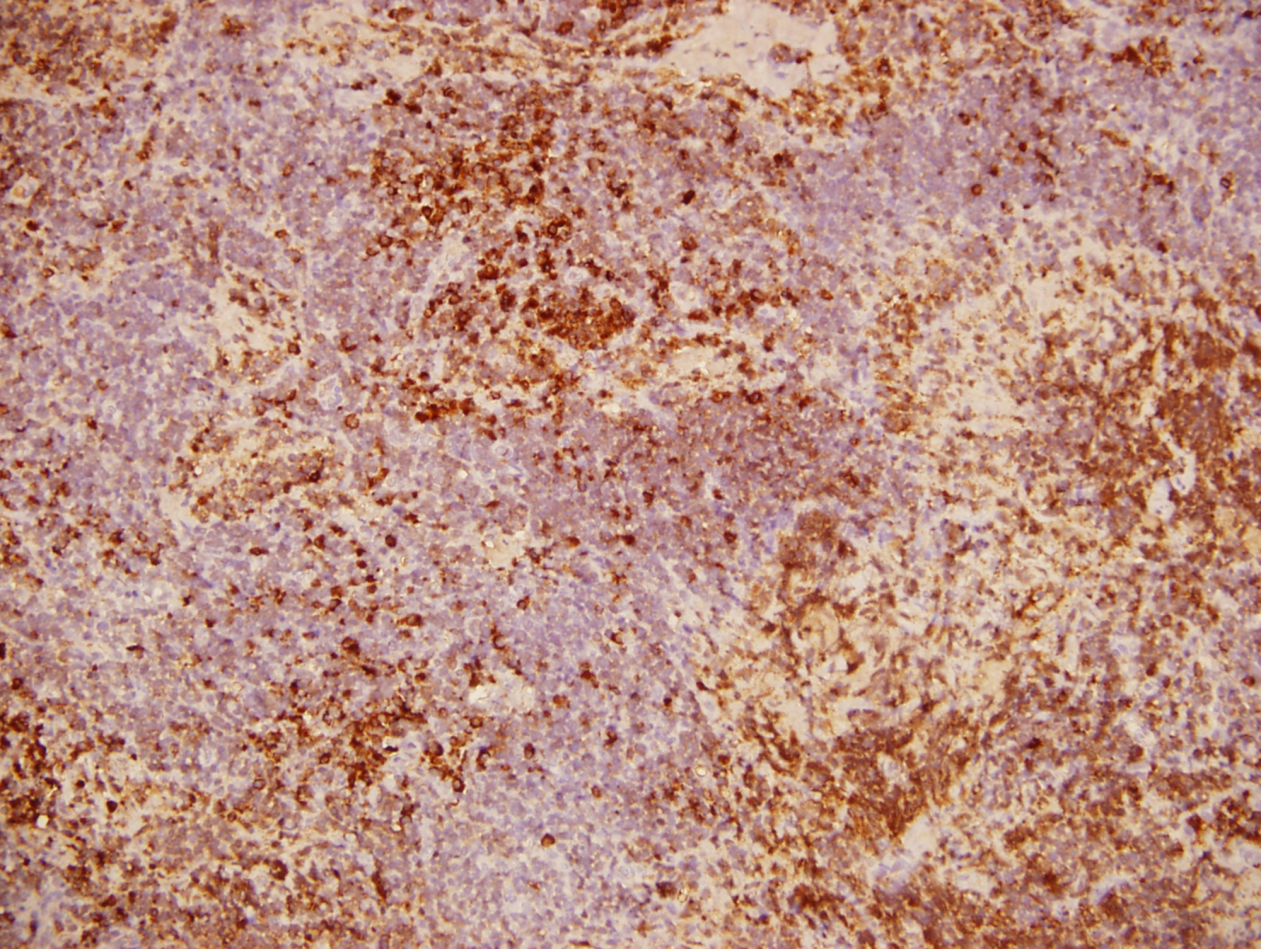
Retroperitoneal adenopathy biopsy (Kappa light chains)

**Figure 5:**
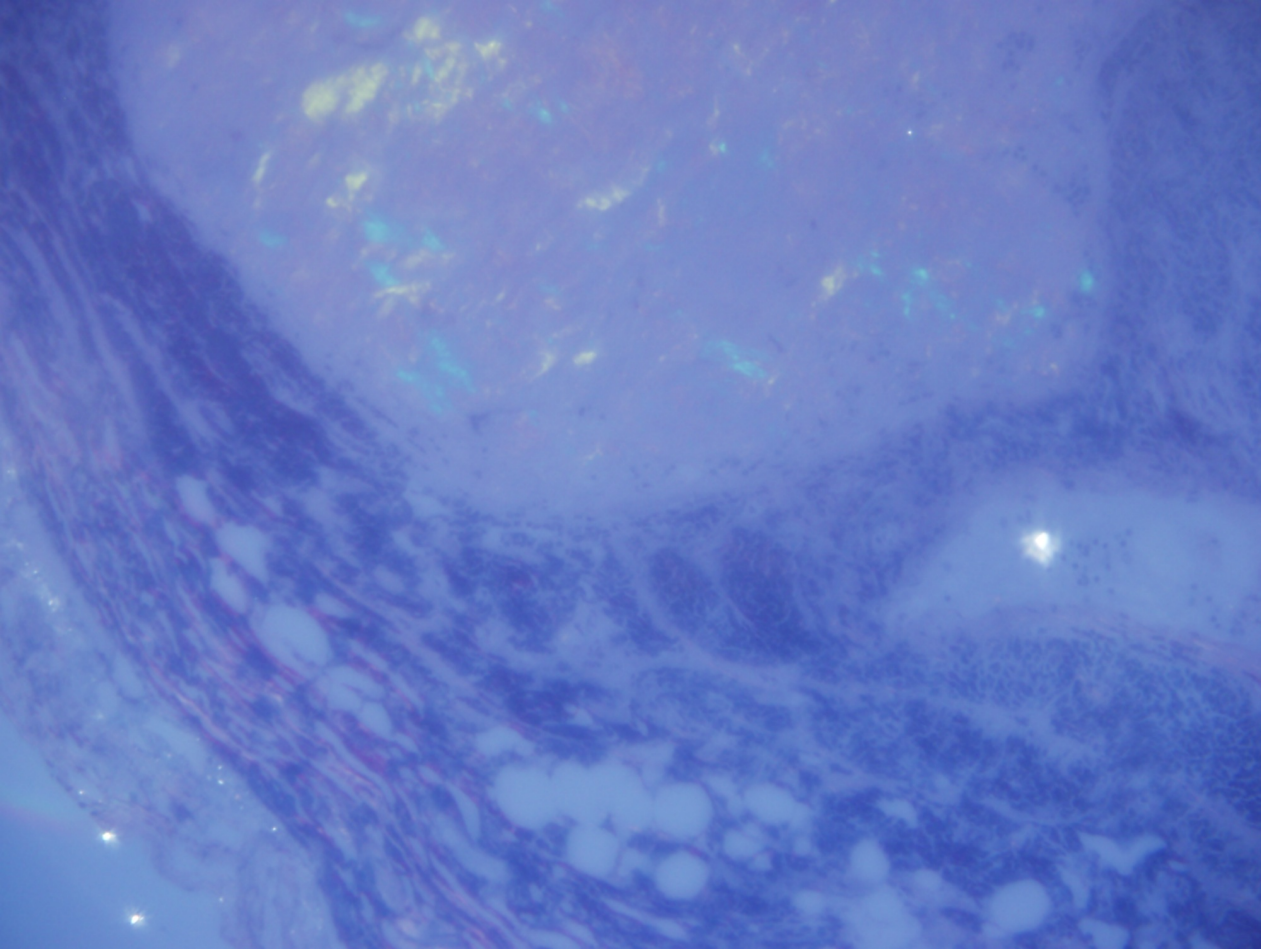
Congo red amyloid stain

A biopsy of retroperitoneal adenopathy was performed by laparoscopy, and the preliminary report showed atypical lymphoid population. The hematologist decided to order studies for monoclonal gammopathy and performed a BM aspiration and biopsy. IgM was elevated (1,465 mg/dL), serum electrophoresis had a monoclonal band in the B2 region, serum immunofixation was positive for monoclonal gammopathy IgM Kappa, serum Kappa free light chain were elevated (578.9 mg/L), and urine immunofixation was positive for Bence Jones Kappa proteins. The final report of the retroperitoneal adenopathy was LPL with Congo red positive for amyloid. The BM aspiration and biopsy were also positive for LPL (Figures 2-5). The diagnosis of WM and amyloid light-chain (AL) amyloidosis was made.

The patient was started on chemotherapy with rituximab, cyclophosphamide, and dexamethasone. She then developed nosocomial pneumonia and was transferred to the intensive care unit. The patient was treated with broad-spectrum antibiotics with requirements of vasopressor support. She recovered from the infection and received the second cycle of chemotherapy. Despite treatment, the chylothorax remained active; therefore, pleurectomy and decortication by thoracoscopy and talc pleurodesis, respectively, were performed.

The response was not the expected with persistence of chylothorax and evidence of new supraclavicular adenopathy. A new CT scan of the thorax and abdomen was performed, which showed signs of progression of the disease. The images also showed a pulmonary embolism, and the patient was started on anticoagulation with low molecular weight heparin. She was briefed by the staff, and given the progression of the disease, it was decided to change the chemotherapy to rituximab, bendamustine, and dexamethasone.

Despite this, the chylothorax showed no improvement; therefore, octreotide was started. A lymphangiography was performed, but there was no evidence of lesions that could undergo embolization. The second cycle of rescue chemotherapy was given one month after the first one. Studies were conducted to evaluate the response to chemotherapy. The levels of IgM, urine protein excretion, and serum Kappa free light chain, as well as evidence of reduction of adenopathies on CT scans, decreased. The option of autologous hematopoietic cell transplantation was proposed, but given the recent pulmonary embolism and the nutritional status, it was deferred. She then received a third cycle of rescue chemotherapy. The evolution began to improve slowly, the production of the chylothorax stopped, and the thoracostomy tube was removed, with no accumulation of fluid. She was discharged with continuation of ambulatory chemotherapy.

## Discussion

Chylothorax refers to the presence of chyle in the pleural space. It is a rare cause of pleural effusion and results from thoracic duct damage with chyle leakage into the pleural space. The etiology can be divided into traumatic and non-traumatic. Thoracic surgery is the leading cause of traumatic chylothorax. Malignancy is the commonest cause of non-traumatic chylothorax, with lymphoma being found in 70% of cases. Pleural fluid triglyceride levels > 110 mg/dL, the presence of chylomicrons, low cholesterol level, and elevated lymphocyte count are diagnostic of chylothorax [5].

WM is an uncommon disease with an annual age-adjusted incidence of WM is 0.38 per 100,000 persons per year. The median age range at diagnosis is 63 to 73 years. It is nearly twice as common in males and whites as compared with females and non-whites [3].

The clinical manifestations of WM develop secondary to direct tumor infiltration and/or effects of IgM monoclonal protein in the blood (Table 1) [3,4]. The most common presenting features are anemia, hyperviscosity-related symptoms, B symptoms, bleeding, and neurological symptoms [2]. Approximately 3% of patients develop AL amyloidosis, and organs that are more commonly affected are the heart, peripheral nerves, kidneys, soft tissues, and liver [4]. Our patient had pulmonary compromise secondary to direct tumor infiltration and AL amyloidosis.

**Table 1:** Clinical manifestations of Waldenström macroglobulinemia IgM, immunoglobulin M

Pathophysiology: frequency	Clinical features
Cell infiltration mediated	
Bone marrow (100%)	Anemia, thrombocytopenia, neutropenia
Extra-medullar hematopoietic tissues (25%)	Lymphadenopathy, hepatomegaly, splenomegaly
Lung (4%)	Cough, dyspnea, nodules or diffuse infiltrates, pleural effusion
Leptomeningeal involvement (rare)	Confusion, seizures, headache, cranial nerve involvement
IgM paraprotein mediated	
Hyperviscosity (30%)	Blurring or loss of vision/diplopia, headache, vertigo, nystagmus, ataxia, oronasal bleeding, confusion, dementia, disturbances of consciousness, stroke, coma, heart failure
IgM-related neuropathy (40%)	Distal and symmetric neuropathy, paresthesias, aching discomfort, dysesthesias or lancinating pains, imbalance and gait ataxia, leg muscle atrophy
Cryoglobulinemia (Asymptomatic in up to 20%; symptomatic ≤ 5%)	Raynaud phenomenon/acrocyanosis, peripheral neuropathy, purpura, skin ulceration or necrosis, arthralgia, glomerulonephritis-related hematuria
Cold agglutinin hemolytic anemia (10%)	Hemolytic anemia
Cell infiltration + IgM paraprotein mediated	
Kidney (4%)	Infiltration of neoplastic cells, Bence Jones proteinuria, light chain cast nephropathy, nephrotic syndrome
Gastrointestinal (4%)	Malabsorption, diarrhea, mucosal bleeding
Skin (3%)	Cutaneous plaques, urticarial skin lesions

Pulmonary involvement represents only 3-5% of cases [4]. The most common radiographic findings are parenchymal infiltrates and pulmonary masses. Isolated pleural effusion is rare and generally serohematic [6]. One study reported 44 patients with confirmed pulmonary disease secondary to WM. Chest X-ray showed pulmonary tumors in 22 patients, with diffuse, reticular, or miliary pulmonary infiltrates in 10 patients; the pulmonary infiltrates occurred together with pleural effusion in 8 patients; and pleural effusion was reported in 19 patients, but only as an isolated finding in 4 patients [7]. In another study that included 217 patients with WM, the pulmonary involvement was a rare manifestation, presenting in 4% of the patients [2]. A retrospective analysis of 985 patients with WM showed that 13 patients had pulmonary involvement, and the main presentations were masses/nodules and pleural effusion [8].

Based on these studies, chylothorax is a very uncommon presentation, with only nine cases reported to date in literature [5,6,9-15]. In three of these cases, chylothorax was the initial manifestation of WM [10-12], whereas in the others, it occurred months or years after the diagnosis of WM. Most of the patients reported were male and between 50 and 82 years of age. One patient died due to an infiltration of LPL cells throughout his entire body [13]. One patient was treated with chemical pleurodesis [6]. One patient was treated with rituximab, oral cyclophosphamide, and dexamethasone, with improvement [14]. One patient was treated with ibrutinib [5]. One patient was treated initially with conservative measures (fasting, total parenteral nutrition, and subcutaneous octreotide); however, the patient presented with recurrence, for which videothoracoscopy was performed, and was managed with a patch and talc pleurodesis, with good response [15]. We did not assess the treatment and outcomes of the other cases.

The treatment of chylothorax involves the following three categories: treatment of the underlying condition, conservative management, and surgical management. Initial conservative measures include drainage of large effusions, nutritional replacement, and diet with low-fat medium-chain triglycerides. Somatostatin and octreotide can be used as adjunct treatments. Surgical therapy is recommended in cases where conservative treatment is not effective. Options include thoracic duct ligation and pleurodesis. In patients with malignancy, where there is no improvement with radiotherapy or chemotherapy, pleurodesis is an alternate option. Other interventions include cannulation and embolization [16]. Our patient had a resistant to chylothorax that required management with diet, second-line chemotherapy, talc pleurodesis, and octreotide; she underwent lymphangiography with no evidence of lesions that could be embolized.

Two mechanisms have been proposed as an explanation of chylothorax in malignancy: pleural infiltration from neoplastic cells and obstruction of the thoracic duct secondary to adenopathies [6]. In our patient, the probable mechanism was obstruction from retroperitoneal adenopathies.

## Conclusions

WM is an uncommon disease, and pulmonary manifestations represent the minority of cases, with few studies reporting chylothorax as a manifestation of WM. Even though it is a rare presentation, it must be taken into consideration within the differential diagnosis of chylothorax so that rapid specific treatment can be initiated and prevent further morbidity. Diagnosis is based on the histopathological confirmation of BM and the detection of any amount of monoclonal IgM protein. All the possible organ damage should be investigated so that treatment can be planned.
